# Comparison of PiCCO and VolumeView: simultaneous measurement in sepsis pig models

**DOI:** 10.1186/cc13333

**Published:** 2014-03-17

**Authors:** H Imahase, S Inoue, Y Sakamoto, T Miyasho, K Yamashita

**Affiliations:** 1 Saga University Hospital, Saga City, Japan; 2 Rakuno Gakuen University, Ebetsu City, Japan

## Introduction

Transpulmonary thermodilution is useful in the critical care. Following PiCCO, VolumeView is now available. Although previous studies concluded that significant correlation was found between VolumeView and PiCCO [1],[2], all experiments and data analysis were done by Edwards Lifescience. The aim of the present study was to compare the new VolumeView with PiCCO to clarify their compatibility and to give a neutral answer.

## Methods

Six pigs (about 10 kg) were used and we made sepsis models by LPS administration. All pigs were instrumented with a right (Pulsio- Cath) and a left (VolumeView) thermomistor-tipped femoral arterial catheter. The central venous catheter was inserted through the right jugular vein. CO, GEDV and EVLW were measured by the two systems.

## Results

**Figure 1:**
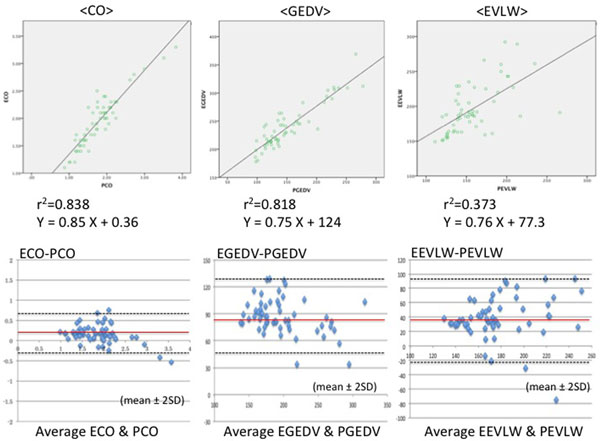
**CO, GEDV, EVLW correlation and Bland-Altman analysis**.

We performed measurements at 57 points. There was a good correlation between the two devices regarding CO and GEDV, but VolumeView showed higher GEDV than PiCCO. Regarding EVLW, there was no significant correlation between two systems. VolumeView showed significantly higher EVLW than PiCCO (Figure 1 and Table 1).

**Table 1:** Explanation of abbreviations

	CO	GEDV	EVLW
PiCCO	PCO	PGEDV	PEVLW
VolumeView	ECO	EGEDV	EEVLW

## Conclusion

Our data analysis showed that PiCCO and VolumeView were not the same. Neither the normal values of two devices nor the rates of change were same. Careful attention should be paid to interpret the data obtained from two systems.
